# Hepatobiliary and pancreatic tuberculosis: A two decade experience

**DOI:** 10.1186/1471-2482-7-10

**Published:** 2007-06-24

**Authors:** Sundeep S Saluja, Sukanta Ray, Sujoy Pal, Manu Kukeraja, Deep N Srivastava, Peush Sahni, Tushar K Chattopadhyay

**Affiliations:** 1 Department of Gastrointestinal Surgery, Room No 1005, PC 1st floor, All India Institute of Medical Sciences, New Delhi, India; 2 Department of Radiology, Room No 68, Ground floor, All India Institute of Medical sciences, New Delhi, India; 3 Department of Pathology, 1st floor, All India Institute of Medical sciences, New Delhi, India

## Abstract

**Background:**

Isolated hepatobiliary or pancreatic tuberculosis (TB) is rare and preoperative diagnosis is difficult. We reviewed our experience over a period two decades with this rare site of abdominal tuberculosis.

**Methods:**

The records of 18 patients with proven histological diagnosis of hepatobiliary and pancreatic tuberculosis were reviewed retrospectively. The demographic features, sign and symptoms, imaging, cytology/histopathology, procedures performed, outcome and follow up data were obtained from the departmental records. The diagnosis of tuberculosis was based on granuloma with caseation necrosis on histopathology or presence of acid fast bacilli.

**Results:**

Of 18 patients (11 men), 11 had hepatobiliary TB while 7 had pancreatic TB. Two-thirds of the patients were < 40 years (mean: 42 yrs; range 19–70 yrs). The duration of the symptoms varied between 2 weeks to 104 weeks (mean: 20 weeks). The most common symptom was pain in the abdomen (n = 13), followed by jaundice (n = 10), fever, anorexia and weight loss (n = 9). Five patients (28%) had associated extra-abdominal TB which helped in preoperative diagnosis in 3 patients. Imaging demonstrated extrahepatic bile duct obstruction in the patients with jaundice and in addition picked up liver, gallbladder and pancreatic masses with or without lymphadenopathy (peripancreatic/periportal). Preoperative diagnosis was made in 4 patients and the other 14 were diagnosed after surgery. Two patients developed significant postoperative complications (pancreaticojejunostomy leak [1] intraabdominal abscess [1]) and 3 developed ATT induced hepatotoxicity. No patient died. The median follow up period was 12 months (9 – 96 months).

**Conclusion:**

Tuberculosis should be considered as a differential diagnosis, particularly in young patients, with atypical signs and symptoms coming from areas where tuberculosis is endemic and preoperative tissue and/or cytological diagnosis should be attempted before labeling them as hepatobiliary and pancreatic malignancy.

## Background

Abdominal tuberculosis (TB) commonly affects the intestinal tract, lymph nodes, peritoneum and solid organs in varying combinations. Up to two-thirds of patients with abdominal TB have abdominal lymphadenopathy or peritoneal disease in addition to intestinal involvement. One-third may have extra-intestinal involvement also [1]. Isolated hepatobiliary or pancreatic TB is rare and the preoperative diagnosis is difficult. The available literature related to hepatobiliary or pancreatic tuberculosis is mostly in the form of case reports or series. Therefore we reviewed our experience with this rare form of abdominal TB in a tertiary health care centre and referral hospital situated in an area endemic for TB.

## Methods

In this retrospective study, the records of 18 patients with proven histological diagnosis of hepatobiliary and pancreatic TB over a period of 20 years (January1986–December 2006) were reviewed. The data pertaining to demographic features, sign and symptoms, duration of illness, imaging (X-ray chest, ultrasonography [US], contrast enhanced CT scan and magnetic resonance imaging [MRI]), side-viewing endoscopy, cytology and/or histopathology, operative findings, therapeutic procedures, postoperative outcome and follow up with anti tubercular therapy (ATT) were obtained. The samples for cytology and/or histology were obtained by US or CT guided aspiration, bile cytology and during exploratory laparotomy. The histological findings of granuloma with caseation necrosis or identification of acid fast bacilli was used as the criteria for diagnosis of tuberculosis.

## Results

Of 18 patients (11 men), 11 had hepatobiliary TB while 7 had pancreatic TB. Of the 11 patients with hepatobiliary TB, 4 had biliary involvement secondary to involvement of the adjacent periportal and pericholedochal lymph nodes. Of the 7 patients with pancreatic TB, 4 had pancreatic involvement secondary to involvement of the adjacent peripancreatic lymph nodes (Table I). The mean age of the patients was 41.7 (range 19–70 years) and two-thirds of them were between third and fourth decade. The duration of symptoms varied between 2 weeks and 2 years (mean: 20 weeks).

**Table 1:** Distribution of patients based on the organ of involvement (n = 18)

Organ	No. of patients
Liver	2
Gallbladder	3
Bile duct	2
Bile duct secondary to periportal lymph node	4
Pancreas alone	3
Pancreatic and peripancreatic lymph node	4

### Clinical presentation

The most common presenting symptom was abdominal pain (*n *= 13) followed by jaundice (*n *= 10), fever (*n *= 9), anorexia (*n *= 9) and weight loss (*n *= 9). Only five (28%) had associated extra-abdominal TB (pulmonary [2], spinal [1] and cervical lymph node TB [2]). Three patients had associated peritoneal (*n *= 2) and splenic involvement (*n *= 1). Physical examination revealed enlarged supraclavicular lymphnode enlargement (SCLN) in 2 patients. The important findings on abdominal examination included hepatomegaly *n *= 9) palpable gallbladder (*n *= 4), gallbladder mass (*n *= 1) and abdominal mass (*n *= 1). The signs and symptoms related to the site of TB are shown in table II. The derangements in laboratory parameters were non specific (Table III).

**Table 2:** Clinical features of patients based on the organ involved.

Symptoms and signs	Liver (n = 2)	Gallbladder (n = 3)	Biliary (n = 6)	Pancreatic (n = 7)	Overall incidence (n = 18)
Abdominal pain	1	2	5	5	13
Jaundice	1	0	6	3	10
Fever	2	1	4	2	9
Anorexia	2	1	3	2	9
Weight loss	2	1	3	3	9
Melaena	1	1	0	0	2
Enlarged Supraclavicular lymph nodes	0	0	0	2	2
Hepatomegaly	1	1	4	3	9
Palpable gallbladder	1	0	1	2	4
Gallbladder mass	0	1	0	0	1
Abdominal mass	0	0	0	1	1

**Table 3:** Results of laboratory investigations of the patients (n = 18)

Investigation	Mean (range)
Haemoglobin (g/dl)	10 (5–13)
Erythrocyte sedimentation rate (mm/hr)	31 (4–50)
Total bilirubin (mg/dl)	5.8 (0.5–25.4)
Aspartate transaminase (IU/L)	447 (94–1637)
Alanine transaminase (IU/L)	95 (26–431)
Alkaline phosphatase (IU/L)	100 (14–422)

### Imaging

Chest X-ray t was normal in all patients except two who had evidence of old, healed pulmonary TB. All patients underwent abdominal US as the first investigation for obstructive jaundice or upper abdominal pain. It showed dilated intrahepatic biliary radicals in 10 patients (lower end block in 6 and hilar or suprapancreatic block in 4). The other findings were a space occupying lesions (SOL) in the liver (*n *= 2), gallbladder mass (*n *= 3), gallbladder polyp (*n *= 1), hyperechogenecity of the gallbladder (*n *= 1) and a pancreatic mass (*n *= 3). Only 2 patients had enlarged periportal and/or peripancreatic lymph nodes on USG.

Abdominal computed tomography (CT) done in all cases, confirmed the findings on US and also showed enlarged periportal, peripancreatic and/or coeliac lymph nodes in 4 patients. Only one patient, who had a pancreatic head mass with enlarged lymph nodes, was suspected to have pancreatic tuberculosis based on CT scan alone. In all others the findings suggested an alternative diagnosis.

Endoscopic retrograde cholangiopancreatography (ERCP) was attempted in 5 patients and bile was aspirated in all patients. In one patient with a hilar stricture, bile cytology demonstrated acid-fast bacilli and the patient was treated with ATT. Magnetic resonance cholangiopancreatography (MRCP) done in 4 patients (hilar block [*n *= 2], postcholecystectomy (*n *= 1) and suspected chelodocholithiasis [*n *= 2]), did not show any mass or filling defect.

### Management

Only 4 patients (22%) could be diagnosed preoperatively, 3 had pancreatic TB and 1 had bile duct TB (presented with hilar stricture). Among 3 patients with pancreatic TB, 1 patient had history of abdominal pain since 2 years and enlarged matted right supraclavicular lymph node on physical examination. Fine needle aspiration cytology (FNAC) from the SC Lymph node showed evidence of TB and a subsequent US-guided FNAC from the pancreatic head mass revealed TB. In the other 2 patients, the diagnosis was made incidentally as CT scan of these patients showed a large, unresectable pancreatic head mass with enlarged para-aortic lymph nodes. Hence, they were considered for palliative chemoradiotherapy. The US-guided FNAC done to confirm the diagnosis of malignancy suggested TB. The patient with a hilar stricture had a history of spinal TB and imaging did not show any mass. He was subjected to ERCP and stenting to decrease the jaundice and a specimen of bile obtained at showed cytological evidence of TB. ATT was started in all four patients. On subsequent follow up, symptoms of these patients resolved completely.

In the remaining 14 patients, a surgical intervention was done based on the working diagnosis. The procedures done included hepatectomy (*n *= 2); (excision of segment VIII and left lateral segmentectomy), cholecystectomy (*n *= 2), extended cholecystectomy (*n *= 1), cholecystectomy, choledochotomy and T-tube drainage (*n *= 2), Roux-en-y hepaticojejunostomy and a frozen section biopsy of the pericholedochal lymph nodes (*n *= 3), Whipple's pancreaticoduodenectomy (*n *= 3) and distal pancreatectomy with splenectomy, choledochojejunostomy and cholecystectomy (*n *= 1). An intraoperative frozen section was sent in 5 patients and suggested TB in 3.

There was no mortality related to surgery. Two patients developed major postoperative complications; one patient following a Whipple's pancreaticoduodenectomy developed a leak from the pancreaticojejunostomy and full thickness wound dehiscence (treated with regular dressing, antibiotics and maintainence of nutrition through feeding jejunostomy), and the other patient who had resection of segment VIII, developed an intra-abdominal abscess requiring percutaneous drainage. Three patients developed ATT-induced hepatotoxicity (elevated level of transaminases [2], jaundice [1]), which required modification in the drugs and dose of ATT. These patients recovered completely and remained asymptomatic on follow up.

The median follow up period was 12 months (9 – 96 months). Two patients developed recurrent jaundice. One patient with bile duct TB who underwent hepatojejunostomy developed recurrent cholestatic jaundice 9 months after surgery. She underwent percutaneous transhepatic drainage for cholangitis and was later taken up for surgical decompression. At re-exploration a kink in the Roux-limb was unraveled to relief the obstruction to biliary outflow. However, the patient continued to deteriorate and succumbed to sepsis and multiple organ dysfunction syndrome 1 year after the primary procedure. The second patient with hepatic TB developed jaundice at 8 years of follow up. On evaluation he was detected to have a single stone in the common bile duct which was removed endoscopically. One patient with pancreatic TB developed an incisional hernia.

## Discussion

In regions with high prevalence of pulmonary TB, abdominal TB is not uncommon. Its prevalence in developing countries has been estimated to be as high as 12% [2]. Although abdominal TB is thought to be frequently associated with active pulmonary TB, evidence of active pulmonary TB has been reported to occur in 6–38% [3] of cases. Only 2 of our patients had evidence of old healed pulmonary TB. Abdominal TB is distinct from pulmonary tuberculosis in that it affects young adults more commonly (mean age in India, 31 years) [4], and is due to ingestion rather than inhalation of pathogen. Following ingestion the bacilli gain access to the gastrointestinal tract where necrotizing granulomas may develop and then spread to the lymphatics affecting any organ in the GI tract, including hepatobiliary and pancreatic tissue [5].

The primary site of TB is usually not evident in most cases of hepatobiliary and pancreatic TB. The liver is affected most commonly as a part of a miliary spread of tuberculosis from lung via hepatic artery [6]. Infection can also spread through the portal vein or lymphatics especially in patients with concomitant TB of the gastrointestinal tract [7]. The infection reaches the gallbladder by either a hematogenous or lymphatic spread from the primary site, by direct involvement and/or secondary involvement from a pre-existing infection in the liver [8]. The much rarer biliary tract infection is again secondary to excretion of acid-fast bacilli from the liver into the bile [9-11] or secondary to involvement of periportal lymph nodes by TB [12-14]. We encountered 2 patients with direct involvement of the bile duct while 4 others had enlarged lymph nodes causing secondary biliary obstruction. Pancreatic TB is uncommon and isolated involvement is rare. [1,15-17] It can be affected either by the hematogenous route in miliary tuberculosis or by direct spread from contiguous lymph nodes.

The **clinical presentation **of hepatobiliary and pancreatic TB is slow and insidious, with non-specific symptoms and signs. In a large review of 300 patients with abdominal TB from India, men and women developed the disease with equal frequency, and although pain was the most common presentation, the symptomatology was variable and nonspecific [18]. In our patients too, abdominal pain was the most common presentation (*n *= 13) associated with non-specific symptoms such as anorexia, weight loss and fever. Presence of active extra-abdominal TB is an important indicator for suspecting a tubercular aetiology in a patient with abdominal complaints. This enabled us to diagnose 2 patients preoperatively and treat with ATT.

Hepatic TB can occur in miliary, nodular and solitary abscess form [19-21]. Series from Philippines [22] and China [23] showed that patients with hepatic TB presented with fever, abdominal pain and hepatomegaly and both patients in this series had similar complaints. In addition one patient presented with melaena, which is a rare manifestation.

Gallbladder TB may present with features of cholecystitis, [24] a gallbladder mass [9] or with obstructive jaundice due to associated enlarged pericholedochal lymph nodes. Three of our patients had TB of the gallbladder; one presented with a mass in the gallbladder, the second with a polyp in the gallbladder and the third with a hemobilia. Similarly, symptomatic biliary tract TB mimics a primary bile duct or gallbladder cancer or even primary sclerosing cholangitis [10,11]. Of the 6 patients with biliary tract TB, the working diagnosis in 5 patients was biliary tract malignancy (gallbladder carcinoma [2], cholangiocarcinoma [2] and periampullary carcinoma [2]. One patient with spinal TB and obstructive jaundice with a block at the confluence of the right and left hepatic ducts was suspected to have biliary TB and was diagnosed preoperatively.

Xia et al. [25] showed in a series of 16 patients with pancreatic TB, abdominal pain (75%), anorexia/weight loss (69%), malaise/weakness (64%), fever (50%) and jaundice (31%) were the common symptoms. Our patients with pancreatic TB had abdominal pain (*n *= 5) as the most common manifestation followed by anorexia, weight loss and jaundice (*n *= 3). These patients mimic carcinoma pancreas or focal pancreatitis [26].

### Imaging

Tubercular liver abscess or tuberculoma may be detected as a SOL on US (hypoechoic lesions with hyperechoic rims and complex masses) and CT (hypodense lesions) [27-29], but only FNAC or a trucut biopsy can confirm the diagnosis. Two of our patients presented with SOL in the liver, the first was diagnosed as infected hydatid cyst and the second as hepatocellular carcinoma based on CT findings. Maglinte et al. [30] reported that more than 50% of patients with hepatic TB had characteristic calcification on X-ray. However, we did not see this finding in our patients.

In patients with gallbladder and bile duct TB, imaging modalities such as US, CT, MRCP and ERCP are useful to define the mass, the level and extent of bile duct obstruction but almost never suggest a diagnosis of TB. Patients with pancreatic TB may show an enlarged pancreas with focal hypodense lesions and irregular borders, most commonly in the head region or enlarged peripancreatic lymph nodes on CT [31]. However these findings are non-specific and may be seen in focal pancreatitis or pancreatic carcinoma.

### Diagnosis

The definitive diagnosis rests on histological and bacteriological evidence of tuberculosis. In view of the nonspecific presentation and imaging appearance of the disease, a high index of suspicion is required to obtain a preoperative diagnosis. However, the diagnosis is usually established at laparotomy, as happened in 14 of 18 of our series. Positive yield of AFB from bile cytology on ERCP is low; only 1 of 5 of our patients had AFB positivity on biliary cytology. Techniques for biopsy include endoscopic US-guided biopsy, CT/US-guided percutaneous biopsy, and surgical biopsy [32]. Mallery et al. had shown that there is no difference in accuracy between these three techniques of pancreatic biopsy [33].

### Treatment

In patients with hepatobiliary and pancreatic TB, once the diagnosis is established, ATT will cure the disease in nearly all patients. However, patients with evidence of biliary obstruction would need either endoscopic or surgical intervention to relieve the obstruction as the ductal narrowing might persist despite treatment with ATT [9,10,34-36]. Most of our patients required a surgical intervention (14/18) because the preoperative diagnosis was not TB but malignancy. The results of surgery in these patients were satisfactory with no postoperative mortality and low morbidity.

## Conclusion

In conclusion, a high index of suspicion in young patients with non-specific symptoms residing in areas with a high prevalence of TB should prompt vigorous attempts at obtaining a preoperative histological or bacteriological diagnosis. In those with no definite diagnosis preoperatively tissue for frozen section should be obtained. However, in those in whom neither a preoperative nor an intraoperative diagnosis of tuberculosis can be made, a surgical excision may be required. This is usually associated with low morbidity and mortality and good long term outcome after adequate ATT.

## Competing interests

The author(s) declare that they have no competing interests.

## Authors' contributions

SS, SP: Acquisition data, analysis and drafted the manuscript, SR acquisition data and helped in drafting DS: Radiologist interpreted imaging, MK: Pathologist provided histological diagnosis PS and TKC: revised it critically. All authors read and approved the final manuscript.

**Table 4:** Details of patients who underwent surgery (n = 14)

No.	Age/sex	Working diagnosis	Per-op findings	Surgical Procedure	Final diagnosis
1	19/F	Haemobilia Infected hydatid cyst	SOL segment VIII 5 × 5 cm containing blood mixed purulent material, CBD dilated with clot	Segment VIII excision with CBD exploration and T-tube drainage.	Hepatic TB
2	60/M	Sol liver ? possibly HCC	SOL in segment 2 and 3, enlarged LN in hepatoduodenal ligament, distended GB, dilated CBD, enlarged spleen with blackish spots	Left lateral segmentectomy with splenectomy and chlecystojejunostomy	Hepatic TB, splenic TB.
3	46/M	Carcinoma GB	Mass in fundus GB, involving segment 4 of liver adherent to duodenum, multiple enlarged LN, CBD 8 mm	Enbloc resection of GB, segment IVB, V, with LN resection antrum and 1^st ^part duodenum, GJ.	GB TB
4	41/M	Haemobilia	GB thick walled with necrosed posterior wall contained clots, CBD not dilated, multiple nodules over parietal, visceral peritoneum	Cholecsytectomy with omental biopsy	GB TB
5	33/F	GB polyp	GB contained polypoidal mass, few enlarged mesenteric LN, tubo-ovarian mass	Cholecystectomy and lymph node biopsy	GB TB
6	35/M	Carcinoma GB with gallstones	GB contracted and thick walled, type I Mirizzi syndrome, proximal CBD dilated with multiple enlarged periportal LN, no stones in GB and CBD.	Cholecystectomy with CBD exploration with T tube drainage with LN biopsy	Periportal LN TB, Chronic cholecystitis
7	70/M	Carcinoma GB	GB thickened with multiple stones, no obvious mass, CBD dilated with 1.5 cm stone, enlarged LN along periportal region	Cholecystectomy with CBD exploration with T tube drainage with Frozen section	Periportal LN TB* chronic cholecystitis
8	46/F	Carcinoma head of pancreas	Large abscess cavity extending from caudate to retroduodenal area, multiple gallstones, CBD dilated 1.5 cm	Exploratory laparotomy + frozen section + cholecystectomy & Roux en y choledochojejeunostomy	Periportal LN TB* Chronic cholecystitis
9	56/F	Cholangiocarcinoma	Mass in mid-CDB circumferential, encasing portal vein and right hepatic artery, multiple LN	Palliative hepaticojejunostomy, LN biopsy, CBD bx	Bile duct TB
10	55/F	Cholangiocarcinoma	Mass CBD with multiple enlarged LN, confluence patent, no liver metastasis	Hepaticojejunostomy with frozen section	Periportal LN TB*bile duct no evidence of malignancy
11	37/M	Pancreatitis, left sided portal hypertension, biliary stricture	Hard shrunken pancreas with multiple collaterals, splenomegaly, dilated CBD	Distal pancreatectomy, splenectomy with choledochojejunostomy	Pancreatic TB
12	60/M	Periampullary carcinoma	Mass periampullary region 2 cm size, dilated CBD, small GB, few LN	Whipple pancreaticoduodenectomy with FJ	Peripancreatic LN TB
13	21/M	Periampullary carcinoma	Bulky head with enlarged peripancreatic, pericholedochal, and paraduodenal LN	Whipple pancreaticoduodenectomy with FJ	Pancreatic and peripancreatic LN TB^•^
14	30/M	Periampullary carcinoma	Mass lower end CBD, 3 × 3 cm infiltrating pancreas with dilated CBD	Whipple pancreaticoduodenectomy with FJ	Pancreatic and peripancreatic LN TB^•^

* Diagnosis obtained from tissue intraoperatively from frozen section

• Tissue sent for frozen section did not provide a diagnosis

**Figure 1:**
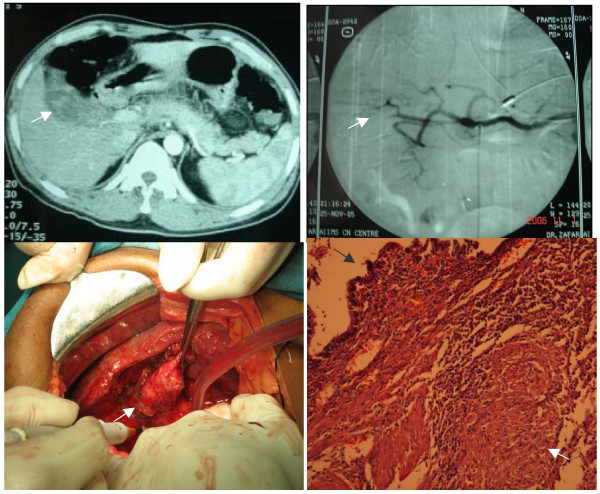
**Case 1 **A 41-year-old man presented with upper gastrointestinal bleed. Endoscopy revealed blood coming out from ampulla **Fig 1a **Axial section of **Dual phase CT scan **shows hyperdensity (white arrow) within the gallbladder **1b Flush aortogram **shows origin of the right hepatic artery directly from the coeliac trunk and spasm in one of the branches (white arrow) **1 c Intraoperative picture **shows multiple tubercles over the gallbladder (white arrow) and liver surface **1 d Histopathology **sections (100×) showing gallbladder mucosa (black arrow) with underlying epithelioid cell granuloma (white arrow). **Final diagnosis: Gall bladder TB**

**Figure 2:**
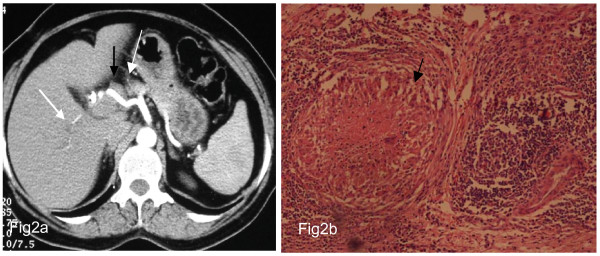
**Case 2 **A 55-year-old woman presented with obstructive jaundice and cholangitis for 2 months. Past history of cholecystectomy done 1 year ago. **Fig 2a CECT **showed mildly dilated intrahepatic radicals (white arrow) with enlarged lymph nodes (black arrow) along the hepatic artery. On exploration mass in CBD with multiple pericholedochal lymph nodes seen. Frozen section of the lymph node showed nectrotizing granuloma. **2b) Histopathology **sections (40×) show epithelioid cell granuloma with central area of caseous necrosis (white arrow). **Final diagnosis: Bile duct TB secondary to periportal lymph nodes**

**Figure 3:**
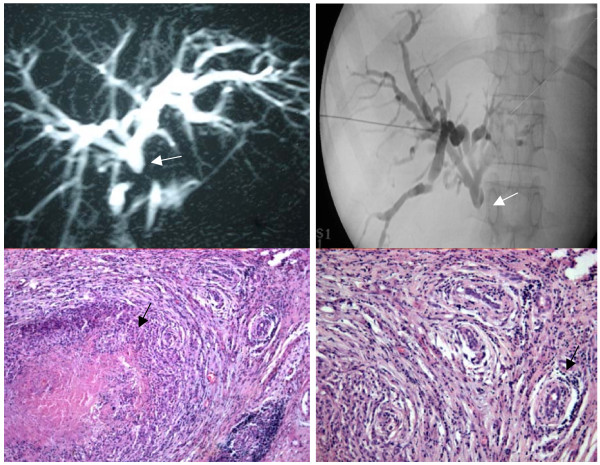
**Case 3 **A 56-year-old woman presented with obstructive jaundice (2 months) associated with cholangitis and previous cholecystectomy (8 years ago). **Fig 3 a) MRCP **done showed dilated intrahepatic radicals with stricture below confluence (white arrow). **3 b) PTBD **done to relieve cholangitis shows stricture below confluence (white arrow). At exploration 2 × 1 cm mass in CBD encasing portal vein with multiple pericholedochal lymph nodes. She underwent palliative hepaticojejunostomy and biopsy of the bile duct and lymph node **3 c) Histopathology **sections examined under low power (40×) show epitheloid cell granuloma with central area of caseous necrosis (black arrow). At the periphery multiple ducts are seen with periductal chronic inflammation and fibrosis. **3 d) **Sections examined under high power (200×) show ducts with dense chronic inflammatory infiltrates comprising of lymphocytes along with periductal fibrosis (black arrow). **Final diagnosis: Biliary tuberculosis**

**Figure 4:**
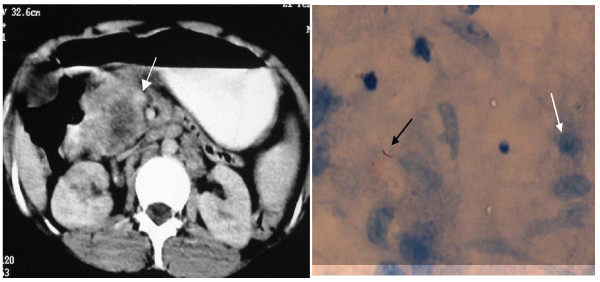
**Case 4 **A 23-year-old woman presented with pain abdomen, weight loss and enlarged supraclavicular lymph nodes for 2 years. **Fig 4a Axial section of CECT **abdomen shows 4 × 2 cm hypodense mass in the region of the head of the pancreas (white arrow) with multiple peripancreatic lymph nodes. **4b) FNAC **from the mass showed acid-fast bacilli (black arrow) surrounded by epithelioid cells (white arrow) **Final diagnosis: Pancreatic tuberculosis**

## Pre-publication history

The pre-publication history for this paper can be accessed here:


